# Prion-dependent proteome remodeling in response to environmental stress is modulated by prion variant and genetic background

**DOI:** 10.1080/19336896.2019.1583041

**Published:** 2019-02-17

**Authors:** Ben Allwein, Christina Kelly, Shaima Kammoonah, Thibault Mayor, Dale M. Cameron

**Affiliations:** a Department of Biology, Ursinus College, Collegeville, PA, USA; b Department of Biochemistry and Molecular Biology, Michael Smith Laboratories, University of British Columbia, Vancouver, British Columbia, Canada

**Keywords:** Yeast prion, prion variant, Sup35, protein misfolding, amyloids, stress response, chronological lifespan, SILAC

## Abstract

A number of fungal proteins are capable of adopting multiple alternative, self-perpetuating prion conformations. These prion variants are associated with functional alterations of the prion-forming protein and thus the generation of new, heritable traits that can be detrimental or beneficial. Here we sought to determine the extent to which the previously-reported ZnCl_2_-sensitivity trait of yeast harboring the [*PSI*^+^] prion is modulated by genetic background and prion variant, and whether this trait is accompanied by prion-dependent proteomic changes that could illuminate its physiological basis. We also examined the degree to which prion variant and genetic background influence other prion-dependent phenotypes. We found that ZnCl_2_ exposure not only reduces colony growth but also limits chronological lifespan of [*PSI*^+^] relative to [*psi*^−^] cells. This reduction in viability was observed for multiple prion variants in both the S288C and W303 genetic backgrounds. Quantitative proteomic analysis revealed that under exposure to ZnCl_2_ the expression of stress response proteins was elevated and the expression of proteins involved in energy metabolism was reduced in [*PSI*^+^] relative to [*psi*^−^] cells. These results suggest that cellular stress and slowed growth underlie the phenotypes we observed. More broadly, we found that prion variant and genetic background modulate prion-dependent changes in protein abundance and can profoundly impact viability in diverse environments. Thus, access to a constellation of prion variants combined with the accumulation of genetic variation together have the potential to substantially increase phenotypic diversity within a yeast population, and therefore to enhance its adaptation potential in changing environmental conditions.

## Introduction

Fungal prions are protein-based epigenetic elements capable of adopting a spectrum of self-propagating conformations that can lead to a variety of heritable phenotypes [1,2]. Nearly one dozen fungal prions have been identified to date [2]. In their native states, many of the fungal prion-forming proteins have roles in gene expression and signaling [2,3], and thus the changes in their functional states that occur upon switching to prion conformations lead to the acquisition of new heritable traits [4,5]. In contrast to the prion conformations of the mammalian prion protein, which are invariably associated with disease [6], these traits can be detrimental or beneficial; indeed, all known fungal prions have the potential to produce beneficial phenotypes in some environments [2]. In many instances, these beneficial traits relate to environmental stress resistance [7–9], and prion formation rates increase significantly during exposure to environmental stress conditions [10–13]. These observations, along with the conservation of prion-forming domains throughout evolution [14] and the widespread existence of prions in wild yeast populations [4,15] have led some researchers to propose that fungal prions serve as a mechanism for propagating altered cellular states that may facilitate adaptation by enhancing survival in rapidly fluctuating environments [4,16,17]. An alternative hypothesis, which suggests that fungal prions are diseases, is based on several observations. For example, one of the earliest studies examining the prevalence of prions in wild yeast strains failed to find either the [*PSI^+^*] or [*URE3*] prions among 70 wild strains examined [18], suggesting these prions have a net deleterious effect on the host cells (notably, the [*RNQ*^+^] prion was found in 11 of the 70 strains, and a later study found [*PSI^+^*] in nearly 1.5% of strains examined and [*RNQ*^+^] in more than 6% of wild strains [4]). The observation of many sick or lethal variants of [*PSI^+^*] or [*URE3*] further supported the notion that prions represent disease states [19], as does the existence of barriers to prion transmission both within and between yeast species [20]. A number of genes have been identified that, when introduced to cells in which they had been deleted, can cure prion variants that arose in their absence, leading to the proposal that yeast possess an array of anti-prion systems [21–23]. Additionally, non-prion roles for some prion-forming domains could explain their evolutionary conservation, and may suggest that prion formation could be an artifact of the non-prion functions of these domains [24–26].

One of the most intensively studied fungal prions is the [*PSI^+^*] prion, which results from alternative conformations of the translation termination factor Sup35 [27–29]. Sequestration of Sup35 into amyloid aggregates compromises its function in translation termination, leading to an increased frequency of nonsense suppression. Read through of stop codons allows cells to sample genetic variation within the 3ʹ untranslated regions (UTRs) of genes to produce proteins with novel C-terminal extensions, which could potentially alter protein function, stability and abundance [16,17]. Since Sup35 has the potential to adopt a spectrum of distinct amyloid structures (prion variants) that impact the strength of the nonsense suppression phenotype [30–32], the phenotypic outcomes of the [*PSI^+^*] prion depend on the unique prion variant present in each strain. Although the overall decrease in translational fidelity and many of the traits it bestows are detrimental [10,17], occasional new beneficial phenotypes conferred by the [*PSI^+^*] prion can ultimately become genetically fixed and thus prion-independent, facilitating rapid adaptation to new environments [12,17].

In an attempt to assess the impact of the [*PSI^+^*] prion on the yeast proteome, we previously employed Stable Isotope Labeling by Amino acids in Cell culture (SILAC)-based quantitative mass spectrometry to compare the proteomes of isogenic [*PSI^+^*] and [*psi^−^*] strains [33]. Only a relatively small number of proteins (~3% of proteins quantified) exhibited significant changes in abundance between the isogenic [*PSI^+^*] and [*psi^−^*] strains in the standard laboratory growth conditions we used in that study. However, since many prion-dependent phenotypes manifest not in standard laboratory growth conditions but rather under conditions of environmental stress [16], more widespread prion-dependent remodeling of the proteome might only become evident in cells exposed to stress. Moreover, since prion-dependent phenotypes are heavily influenced by the genetic background of the strain [16], any prion-dependent changes in protein expression that occur during environmental stress are likely to be idiosyncratic to each strain.

In their seminal study examining [*PSI^+^*]-dependent phenotypes in multiple genetic backgrounds across more than 150 growth conditions, True and Lindquist [16] observed constellations of prion-dependent phenotypes unique to each of the seven genetic backgrounds they studied. Only one environmental condition – growth on agar medium supplemented with 5 mM ZnCl_2_ – consistently conferred a fitness advantage to [*psi*^−^] cells among all genetic backgrounds tested, suggesting that altered zinc metabolism may be a universal feature of [*PSI*^+^] cells. We therefore sought to determine the extent to which the previously-reported ZnCl_2_-sensitivity trait of strains harboring the [*PSI*^+^] prion is modulated by genetic background and prion variant. We found [*PSI*^+^] strains to be sensitive to ZnCl_2_ relative to isogenic prion-free [*psi*^−^] strains, with reduced colony growth and chronological lifespan. The degree of sensitivity was influenced by prion variant and genetic background, and quantitative proteomic analysis of cells exposed to ZnCl_2_ during growth indicates that [*PSI*^+^] cells exhibit increased cell stress and reduced energy metabolism compared to [*psi*^−^] cells. Finally, we show that prion-dependent phenotypes and changes in protein abundance are profoundly influenced by prion variant and genetic background, thus enhancing phenotypic diversity within a population and potentially providing a mechanism for enhancing survival in fluctuating environments.

## Results

### ZnCl_2_ significantly reduces colony size of [PSI^+^] relative to isogenic [psi^−^] yeast

**Figure 1.**
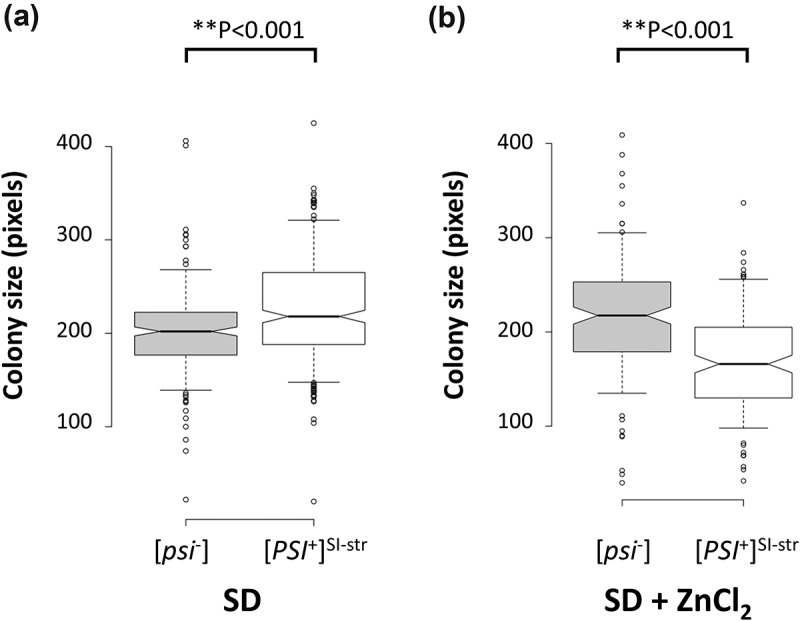
The [*PSI*^+^] prion confers sensitivity to ZnCl_2_. Median colony size for an isogenic pair of [*psi*^−^] and [*PSI*^+^]^SI-str^ strains following growth on SD medium in the absence (a) or presence (b) of 5 mM ZnCl_2_. Colonies were incubated for ~72 hours on SD agar or ~96 hours on SD agar + ZnCl_2_. Center lines show the medians; box limits indicate the 25th and 75th percentiles as determined by R software; whiskers extend to 5th and 95th percentiles, outliers are represented by dots. Notches represent the 95% confidence interval for each median. Non-overlapping notches give ~95% confidence that two medians differ. **p < 0.001 (Z-test). n = 236, 326, 166, 161 sample points.

To confirm that [*PSI*^+^] cells are more sensitive to ZnCl_2_ exposure compared to isogenic [*psi*^−^] cells, we used an S288C-based strain we previously constructed [33] with an induced [*PSI*^+^] variant that produces a strong nonsense-suppression phenotype (determined by the *ade1-14*-based colony color assay), hereafter referred to as [*PSI*^+^]^SI-str^ (for S288C Induced strong variant). First, we cured the strain of the [*PSI*^+^] prion by passage on medium containing 5 mM GuHCl to generate an isogenic [*psi*^−^] strain. We then confirmed that the [*psi*^−^] strain has a fitness advantage compared to the isogenic [*PSI*^+^]^SI-str^ strain by measuring colony size on synthetic defined (SD) agar supplemented with 5 mM ZnCl_2_. While the [*PSI*^+^]^SI-str^ strain produced significantly larger colonies than the isogenic [*psi*^−^] strain on SD agar in the absence of ZnCl_2_ (Figure 1(a)), the opposite was observed in the presence of ZnCl_2_ (Figure 1(b)). Notably, we did not detect any fitness advantage for [*psi*^−^] cells when grown in liquid SD medium supplemented with 5 mM ZnCl_2_ (data not shown), and thus the ZnCl_2_-sensitivity trait for [*PSI*^+^]^SI-str^ may be confined to growth on solid media.

### Exposure to ZnCl_2_ reduces chronological lifespan of [PSI^+^]^SI-str^ cells to a greater extent than isogenic [psi^−^] cells

**Figure 2.**
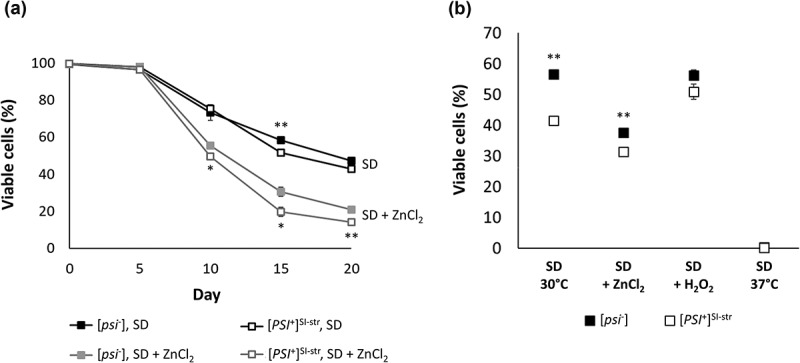
(a). Exposure to ZnCl_2,_ reduces chronological lifespan of [*PSI*^+^]^SI-str^ cells to a greater extent than isogenic [*psi*^−^] cells. (a). Viability was assessed by PI staining followed by flow cytometry over a 20-day time-course on SD agar with and without 5mM ZnCl_2_. Viability on ZnCl_2_ was reduced for both [*psi*^−^] and [*PSI*^+^]^SI-str^ cells; however, [*PSI*^+^] cells lost viability more rapidly than [*psi*^−^] cells. Error bars indicate SEM, n = 3 independent biological replicates per strain. * p < 0.05, ** p < 0.001 (student’s t-test). (b). There is no significant difference in 15-day viability between [*PSI*^+^]^SI-str^ and isogenic [*psi*^−^] exposed to H_2_O_2_ or at 37°C. The [*PSI*^+^]^SI-str^ strain exhibits reduced 15-day viability compared to the isogenic [*psi*^−^] strain on SD and SD + ZnCl_2_ at 30°C, but no significant change in viability on SD agar supplemented with H_2_O_2_ or on SD at 37°C. Error bars indicate SEM, n = 3–4 independent biological replicates per strain. * p < 0.05, ** p < 0.001 (student’s t-test).

The reduction in fitness as measured by colony size may be indicative of a reduction in lifespan and viability for the S288C [*PSI*^+^]^SI-str^ cells when exposed to ZnCl_2_. We therefore asked whether exposure to ZnCl_2_ differentially affects chronological lifespan in S288C [*PSI*^+^]^SI-str^ and isogenic [*psi*^−^] cells. We assessed viability of cells in colonies grown on SD agar with and without 5 mM ZnCl_2_ over a 20-day time-course by staining cells with propidium iodide (PI) and measuring the fraction of inviable cells by flow cytometry. We found that ZnCl_2_ reduces viability in both [*psi*^−^] and [*PSI*^+^]^SI-str^ cells; however, by the end of the time-course we observed a small but significant exacerbation of the ZnCl_2_-sensitivity phenotype in [*PSI*^+^]^SI-str^ cells (Figure 2(a)). By day 20 there was no significant difference in the relative viability of [*psi*^−^] and [*PSI*^+^] cells in the absence of ZnCl_2_, yet a nearly 50% reduction in viability of [*PSI*^+^] relative to [*psi*^−^] cells in the presence of ZnCl_2_. Taken together, our data indicate that the reduction in colony growth rate and viability induced by exposure to ZnCl_2_ on solid media is modulated by the prion status of the yeast cells.

Is the [*PSI*^+^]^SI-str^ strain simply less fit than the isogenic [*psi*^−^] strain under all stress conditions? To test if this is the case, we examined whether the [*PSI*^+^]^SI-str^ prion confers reduced viability in two other stress conditions: mild heat stress, and mild oxidative stress. Since the largest differences in viability between the isogenic [*psi*^−^] and [*PSI*^+^] strains (both with and without ZnCl_2_) were observed at 15 days (Figure 2(a)), we examined 15-day viability in SD supplemented with 100 µM H_2_O_2_, and in SD at 37°C (Figure 2(b)). In contrast to growth at 30°C in SD and SD + ZnCl_2_, no significant differences in 15-day survival were observed between isogenic S288C [*psi*^−^] and S288C [*PSI*^+^]^SI-str^ strains in the presence of H_2_O_2_ or at 37°C (Figure 2(b)). Thus, the sensitivity phenotype of the [*PSI*^+^]^SI-str^ strain when exposed to ZnCl_2_ is not a general stress sensitivity phenotype, but rather it is specific for this environmental condition.

### ZnCl_2_ enhances expression of stress response proteins and reduces expression of proteins involved in energy metabolism in [PSI^+^] cells relative to isogenic [psi^−^] cells

**Figure 3.**
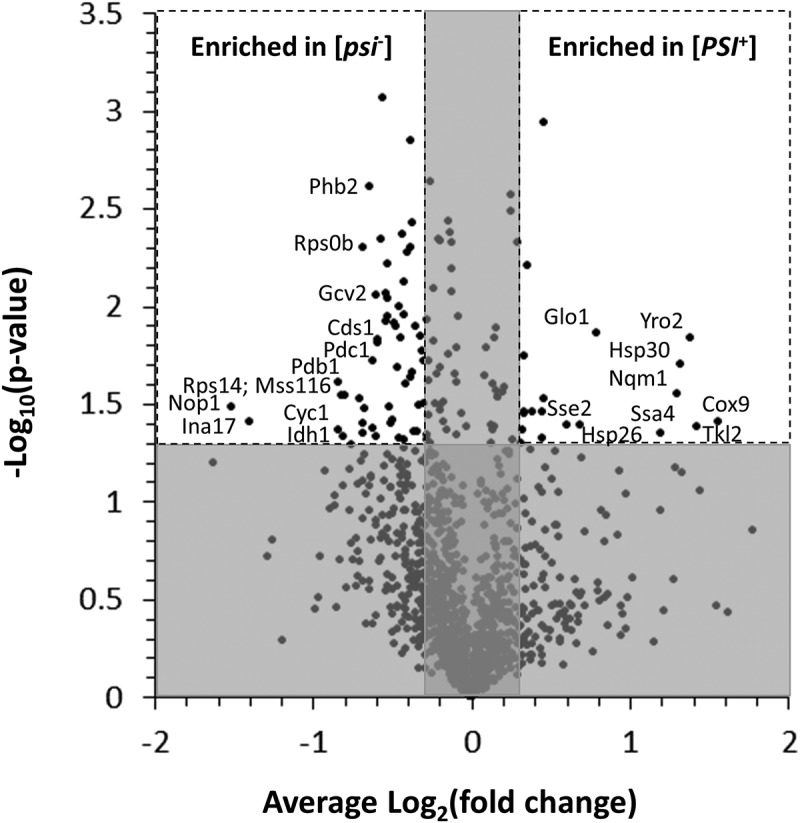
Proteins with significantly increased or decreased abundance in the [*PSI*^+^]^SI-str^ strain relative to the isogenic [*psi*^−^] strain when grown on SD agar supplemented with 5 mM ZnCl_2_. The SILAC approach followed by quantitative mass spectrometry was utilized to examine the relative abundance of proteins in the [*PSI*^+^]^SI-str^ strain relative to an isogenic [*psi*^−^] strain. Proteins with <25% change in abundance (vertical shading) or for which changes in abundance were not significant (P > 0.05; horizontal shading) are indicated. Examples of proteins exhibiting significant changes in abundance of >50% (increase or decrease in [*PSI*^+^]^SI-str^) are identified by name (see Table S1 for a complete list).

The reduction in fitness and viability for the [*PSI*^+^]^SI-str^ strain relative to the isogenic [*psi*^−^] strain when exposed to ZnCl_2_ suggests that [*PSI*^+^]^SI-str^ cells experience more stress than [*psi*^−^] cells in that environment. To determine whether the proteome of [*PSI*^+^]^SI-str^ cells is remodeled differently than that of isogenic [*psi*^−^] cells following growth in the presence of ZnCl_2_, we used SILAC (Stable Isotope Labeling by Amino acids in Cell culture) followed by quantitative mass spectrometry to detect prion-dependent changes in protein abundance in colonies growing on SD agar supplemented with 5 mM ZnCl_2_. We collected data for 1267 proteins (~ one quarter of the proteome) that were quantified in at least two of the three experiments using the Perseus platform integrated to MaxQuant (Table S1). Of those 1267 proteins, the levels of 37 and 87 were significantly increased or decreased in the [*PSI*^+^]^SI-str^ strain in comparison to the isogenic [*psi*^−^] strain, respectively (Figure 3), representing changes in abundance for ~10% of the proteins quantified. Notably, we observed more than two-fold enrichment of some proteins in [*PSI*^+^] cells, including the heat shock-induced Hsp30 and the acid stress response Yro2, whereas the Nop1 histone glutamine methyltransferase, and the Ina17 F1F0 ATPase synthase peripheral stalk assembly factor, were both reduced by more than 50% (Figure 3).

Consistent with the slower growth and reduced viability in [*PSI*^+^] cells compared to [*psi*^−^] cells exposed to ZnCl_2_, proteins involved in stress responses (protein folding) were significantly more abundant (with >25% change in abundance) in [*PSI*^+^]^SI-str^ cells relative to isogenic [*psi*^−^] cells (Table 1), while proteins involved in energy metabolism were significantly less abundant (Table 2). Components of the translation machinery were also less abundant in [*PSI*^+^]^SI-str^ cells, though the false discovery rate (FDR) for this category was >0.05.

**Table 1.** GO-Slim enrichments (biological process) for proteins exhibiting >25% increased abundance in the [*PSI*^+^]^SI-str^ strain relative to the isogenic [*psi*^−^] strain following exposure to ZnCl_2_. Colonies of S288C [*psi*^−^] and [*PSI*^+^]^SI-str^ were grown for ~ 90 hours on SILAC-labeled SD agar with or without 5 mM ZnCl_2,_ then proteins were isolated and subjected to mass specrometry analysis. PANTHER Overrepresentation Test with annotation data set GO-Slim Biological Process using Fisher’s Exact Test with FDR multiple test correction. Only GO-Slim categories for which p < 0.05 are shown; shading indicates categories with a FDR>0.05.

PANTHER GO-Slim Biological Process	Genes annotated in reference list (#)	Genes annotated in analyzed list (#)	Genes expected in analyzed list (#)	Fold enrichment	Raw P value	FDR
protein folding	50	4	0.14	28.3	1.30E-05	2.24E-03
response to abiotic stimulus	37	2	0.1	19.12	5.24E-03	4.51E-01
pentose-phosphate shunt	10	1	0.03	35.38	3.06E-02	8.77E-01
phosphate-containing compound metabolic process	622	5	1.76	2.84	2.65E-02	9.11E-01
response to stress	219	3	0.62	4.85	2.31E-02	9.94E-01

**Table 2.** GO-Slim enrichments (biological process) for proteins exhibiting >25% decreased abundance in the [*PSI*^+^]^SI-str^ strain relative to the isogenic [*psi*^−^] strain following exposure to ZnCl_2_. Colonies of S288C [*psi*^−^] and [*PSI*^+^]^SI-str^ were grown for ~ 90 hours on SILAC-labeled SD agar with or without 5 mM ZnCl_2,_ then proteins were isolated and subjected to mass spectrometry analysis. PANTHER Overrepresentation Test with annotation data set GO Biological Process Complete using Fisher’s Exact Test with FDR multiple test correction. Only GO-Slim categories for which p < 0.05 are shown; shading indicates categories with a FDR>0.05.

PANTHER GO-Slim Biological Process	Genes annotated in reference list (#)	Genes annotated in analyzed list (#)	Genes expected in analyzed list (#)	Fold enrichment	Raw P value	FDR
coenzyme metabolic process	76	6	0.58	10.41	3.05E-05	2.63E-03
generation of precursor metabolites and energy	105	7	0.8	8.79	1.78E-05	3.06E-03
tricarboxylic acid cycle	20	3	0.15	19.77	6.41E-04	3.67E-02
metabolic process	2118	26	16.07	1.62	3.94E-03	1.69E-01
primary metabolic process	1787	22	13.56	1.62	1.06E-02	2.29E-01
glycolysis	21	2	0.16	12.55	1.27E-02	2.43E-01
translation	173	5	1.31	3.81	1.05E-02	2.57E-01
mitochondrial translation	16	2	0.12	16.48	7.88E-03	2.71E-01
cellular amino acid metabolic process	172	5	1.3	3.83	1.02E-02	2.94E-01

### Prion variant and genetic background profoundly influence viability in diverse environments

**Figure 4.**
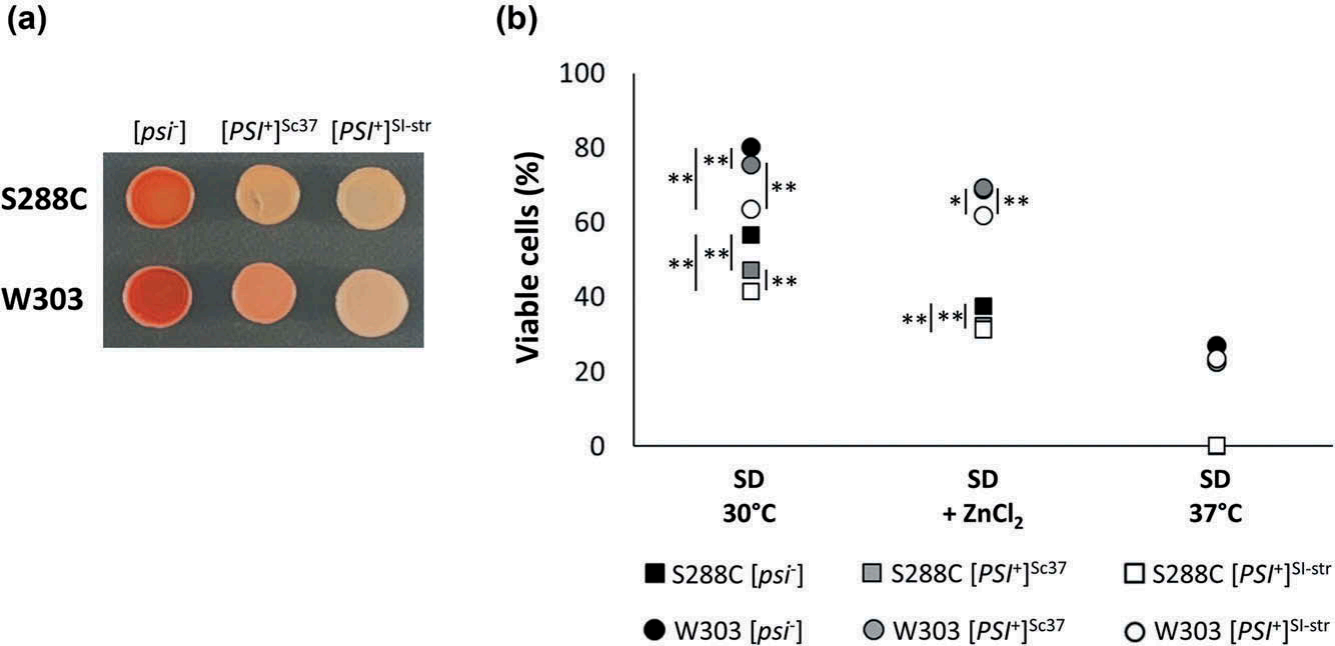
Viability following 15-day incubation in a variety of environmental conditions is profoundly influenced by genetic background and prion variant. (a) Color phenotypes of strong [*PSI*^+^]^SI-str^ and weak [*PSI*^+^]^Sc37^ prion variants in S288C and W303 genetic backgrounds following growth on ¼YEPD agar medium. (b) 15-day viability for S288C and W303 [*psi*^−^] and [*PSI*^+^] prion variants grown on SD at 30°C, SD+ZnCl_2_ (5 mM), SD+H_2_O_2_ (100 µM), and SD at 37°C. Error bars indicate SEM, n = 3–4 independent biological replicates per strain. * p < 0.05, ** p < 0.001 (student’s t-test); statistical significance symbols on the left side of a data marker refer to comparisons between a [*PSI*^+^] strain and the isogenic [*psi*^−^] strain, whereas those on the right side of a data marker refer to comparisons between [*PSI*^+^] variants.

Is the prion-dependent reduction in fitness and lifespan on SD agar supplemented with ZnCl_2_ modulated by genetic background and prion variant? Previous studies [16] found ZnCl_2_ sensitivity to be a universal trait among seven genetic backgrounds tested. We used an established protein transformation protocol [34] to introduce two different [*PSI*^+^] prion variants (the [*PSI*^+^]^SI-str^ variant used here for our SILAC experiments, and the previously described [*PSI*^+^]^Sc37^ weak variant [30,31,35]) into two genetic backgrounds (S288C and W303; Figure 4(a)) and examined 15-day survival in each strain. Importantly, since the [*PSI*^+^]^SI-str^ strain used for the SILAC experiments also exhibited the [*PIN*^+^] phenotype (for [*PSI*^+^]-Inducibility, commonly due to the [*RNQ*^+^] prion), we eliminated any possible effects attributable to [*PIN*^+^] by transforming [*psi*^−^][*rnq*^−^] strains with the [*PSI*^+^] prion variants to generate sets of isogenic S288C or W303 strains that are [*psi*^−^][*rnq*^−^], [*PSI*^+^]^Sc37^[*rnq*^−^], or [*PSI*^+^]^SI-str^[*rnq*^−^]. We found that the extent of ZnCl_2_ sensitivity varied considerably between the S288C and W303 genetic backgrounds (Figure 4(b)). Indeed, the W303 strains all exhibited greater survival on SD supplemented with ZnCl_2_ compared to the S288C strains. We also observed survival differences between strains in other growth conditions. For example, the S288C strains were only marginally viable after 15 days at 37°C, whereas approximately one quarter of W303 cells survived. Furthermore, we observed significant differences in viability based on prion status in both the presence and absence of ZnCl_2_. For example, at 30°C in the absence of ZnCl_2_, in both genetic backgrounds we observed a consistent relationship between survival and prion strength, with [*psi*^−^] cells exhibiting the greatest viability, strains with the stronger [*PSI*^+^]^SI-str^ variant exhibiting the lowest viability, and strains with the weaker [*PSI*^+^]^Sc37^ variant showing an intermediate level of viability. Conversely, in the presence of ZnCl_2_, the effect of prion variant was dependent on the genetic background: S288C strains with either prion variant exhibited equally reduced survival compared to the isogenic [*psi*^−^] strain, whereas in W303 only the stronger [*PSI*^+^]^SI-str^ variant impacted survival. Thus, genetic background, prion variant, and environment all interact to influence cell survival.

### Prion-dependent changes in protein abundance are modulated by prion variant and by genetic background

**Figure 5.**
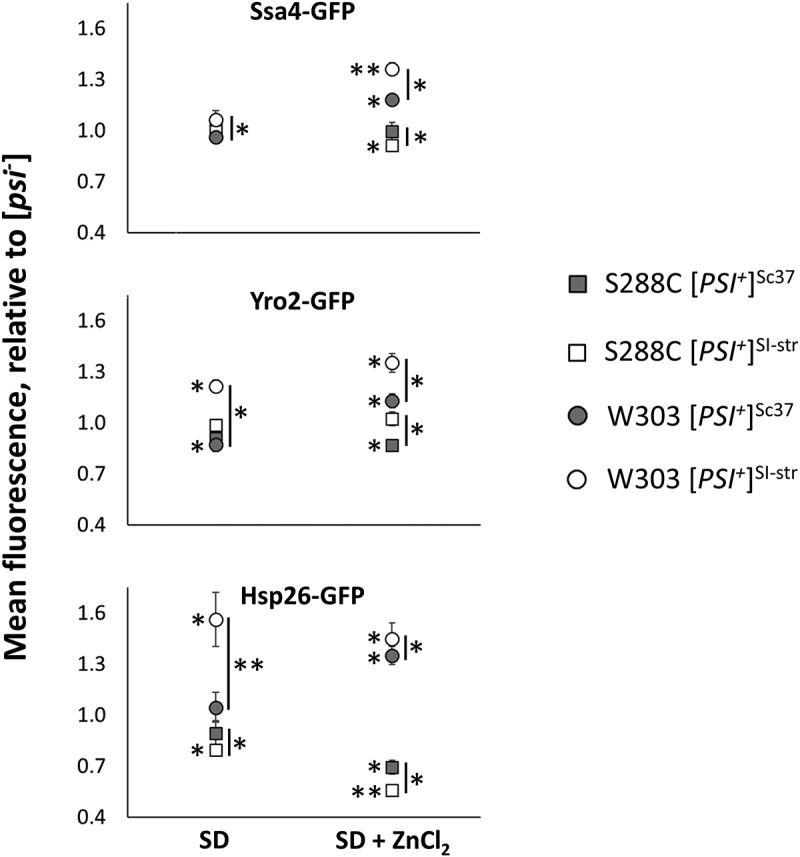
Prion-dependent changes in protein abundance associated with growth on SD agar supplemented with ZnCl_2_ is modulated by prion variant and genetic background. Protein abundance was assessed by measuring fluorescence intensity of GFP-tagged Ssa4, Hsp26, and Yro2 by flow cytometry in S288C and W303 [*psi*^−^][*rnq*^−^], [*PSI*^+^]^Sc37^[*rnq*^−^], and [*PSI*^+^]^SI-str^[*rnq*^−^] strains. Values represent mean fluorescence of each strain (50,000 cells) relative to the isogenic [*psi*^−^] strain in each condition. Error bars indicate SEM, n = 3 independent biological replicates per strain. * p < 0.05, ** p < 0.001 (student’s t-test); statistical significance symbols on the left side of a data marker refer to comparisons between a [*PSI*^+^] strain and the isogenic [*psi*^−^] strain, whereas those on the right side of a data marker refer to comparisons between [*PSI*^+^] variants.

The substantial impact of genetic background and prion variant on survival phenotypes (Figure 4) could, in part, be mediated through prion-dependent remodeling of the proteome in response to environmental stress. To investigate the influence of these factors on protein abundance we examined three proteins identified by our SILAC analysis as exhibiting differential expression in the S288C [*PSI*^+^]^SI-str^[*PIN*^+^] strain relative to the isogenic S288C [*psi*^−^][*PIN*^+^] strain: Ssa4, Yro2, and Hsp26 (all of which exhibited increased abundance in S288C [*PSI*^+^]^SI-str^[*PIN*^+^] compared to [*psi*^−^][*PIN*^+^] in our SILAC analysis; Figure 3). To quantitatively examine protein abundance we introduced C-terminal GFP tags onto Ssa4, Yro2 or Hsp26 in [*rnq*^−^] S288C and W303 strains containing the strong [*PSI*^+^]^SI-str^ or weak [*PSI*^+^]^Sc37^prion variants, then cured each GFP^+^ [*PSI*^+^] strain by passage on medium containing 5 mM GuHCl to generate isogenic [*psi*^−^] strains. We then used flow cytometry to examine relative protein abundance for Ssa4-GFP, Yro2-GFP, and Hsp26-GFP in these sets of isogenic [*psi*^−^][*rnq*^−^], [*PSI*^+^]^Sc37^[*rnq*^−^], or [*PSI*^+^]^SI-str^[*rnq*^−^] strains grown on agar medium with or without 5 mM ZnCl_2_. The use of GFP intensity as a quantitative reporter for protein abundance is well documented in the literature [36,37]. Prion-dependent changes in protein abundance were found to be modulated by prion variant and by genetic background (Figure 5). For all three proteins, there were more significant changes in abundance in [*PSI*^+^] strains (relative to the isogenic [*psi*^−^] strains) in the presence of ZnCl_2_ than in its absence. The magnitudes of the prion-dependent changes in protein abundance were typically greater in the W303 genetic background. In addition, for the W303 strains, the strong [*PSI*^+^]^SI-str^ prion variant always produced the greatest change in protein abundance (relative to the isogenic [*psi*^−^] strain), though this was not the case in the S288C genetic background. For example, we found no significant difference in the relative abundance of Ssa4-GFP between the S288C [*psi*^−^] strain and either S288C [*PSI*^+^] strain in the absence of ZnCl_2_ (and only a small difference between [*PSI*^+^] variants in the W303 background), yet in the presence of ZnCl_2_ we observed reduced abundance of Ssa4-GFP in the S288C [*PSI*^+^]^SI-str^ strain relative to the S288C [*psi*^−^] and [*PSI*^+^]^Sc37^ strains. In the W303 background, both prion variants resulted in significant increases in abundance of Ssa4-GFP relative to the isogenic [*psi*^−^], though the magnitude of the increase was significantly greater for the [*PSI*^+^]^SI-str^ prion variant. In contrast, for Yro2-GFP abundance in S288C strains, only the weak prion variant produced a significant change in abundance relative to the [*psi*^−^] and [*PSI*^+^]^SI-str^ strains in the presence of ZnCl_2_. For Hsp26-GFP, in the absence of ZnCl_2_ the weak [*PSI*^+^]^Sc37^ variant did not produce any change in protein abundance relative to the isogenic [*psi*^−^] strain in the W303 background, whereas the strong [*PSI*^+^]^SI-str^ variant resulted in a large increase in Hsp26-GFP abundance. In contrast, in the presence of ZnCl_2_ the W303 [*PSI*^+^]^Sc37^ strain exhibited a high abundance of Hsp26-GFP relative to the isogenic [*psi*^−^] strain, though not as high as the W303 [*PSI*^+^]^SI-str^ strain. It is also noteworthy that although the W303 [*PSI*^+^]^SI-str^ strains exhibited significant increases in the abundance of all three proteins relative to the isogenic [*psi*^−^] strains in the presence of ZnCl_2_ (consistent with the findings of our SILAC experiment), this was not the case for the S288C [*PSI*^+^]^SI-str^ strains, which exhibited either no change (Yro2-GFP) or decreased abundance (Ssa4-GFP and Hsp26-GFP) relative to the isogenic [*psi*^−^] strains. Since the SILAC experiments were conducted in the S288C background, the inconsistencies in results between the SILAC and flow cytometry-based approaches are likely attributable to two important differences in the strains we employed for each approach. First, and most notably, the presence of the C-terminal GFP tag in the strains used for the flow cytometry-based approach (which also displaced the 3ʹ UTR of the assessed alleles) may influence protein stability and/or stop codon read through frequencies, and thus impact abundance. Secondly, the strains used in the SILAC experiment also exhibited the [*PIN*^+^] phenotype, whereas the strains used for flow cytometry were all cured to [*pin*^−^] prior to transformation with each [*PSI*^+^] variant. However, despite these inconsistencies, our results clearly indicate that both genetic background and prion variant can have a profound impact on prion-dependent changes in protein abundance.

## Discussion

Yeast prions can produce a range of phenotypes that vary depending on environment and on the genotype of the strain. The [*PSI^+^*] prion results in elevated levels of nonsense suppression, and thus has the potential to remodel the proteome to produce new traits. Our previous proteomic analysis, however, identified only relatively small changes between the proteomes of isogenic [*PSI^+^*] and [*psi^−^*] strains in standard laboratory growth conditions [33]. One [*PSI*^+^]-dependent trait, sensitivity to ZnCl_2_, was previously found to be exhibited in all genetic backgrounds examined [16], suggesting that altered zinc metabolism may be a universal feature of [*PSI*^+^] cells. We therefore examined the extent to which this phenotype, and other prion-dependent phenotypes, are influenced by genetic background and prion variant. We found that the ZnCl_2_-sensitivity trait of [*PSI*^+^] cells is indeed modulated by genetic background and prion variant. Quantitative proteomic analysis of cells grown in the presence of ZnCl_2_ identified significant prion-dependent changes in abundance for ~10% of proteins quantified, with [*PSI*^+^] cells exhibiting significant enrichment of stress response proteins and significant reduction of proteins involved in energy metabolism. More generally, we found that prion variant and genetic background profoundly influenced prion-dependent phenotypes and changes in protein abundance.

Our quantitative proteomic analysis revealed that relative to isogenic [*psi*^−^] cells, [*PSI*^+^]^SI-str^ cells exposed to ZnCl_2_ are enriched for heat shock proteins and exhibit reduced abundance of proteins involved in energy metabolism. These findings are consistent with the activation of stress responses and reduced growth in [*PSI*^+^] cells compared to [*psi*^−^] cells exposed to ZnCl_2_, and with previous studies suggesting that the [*PSI*^+^] prion can lead to cell stress and enhance expression of heat shock proteins [38]. Whether the same subsets of proteins would exhibit altered abundance in other genetic backgrounds, or for different [*PSI*^+^] variants, is unclear; however, our flow cytometry analysis of Ssa4-GFP, Hsp26-GFP, and Yro2-GFP indicate that prion-dependent changes in protein abundance that occur when cells are grown in the presence of ZnCl_2_ are modulated by these factors (Figure 5). Since the phenotypic strength of the nonsense suppression phenotype of [*PSI*^+^] is influenced by prion variant [30–32], the variant-dependent changes in protein abundance we observed are likely due to differences in stop codon read-through frequencies. Moreover, since the identity of the stop codon and the sequence context surrounding it impacts the frequency of nonsense suppression [39], the impact of the [*PSI*^+^] prion on gene expression will vary from gene to gene. This natural variation in [*PSI*^+^]-dependent effects on gene expression was not captured by our flow cytometry-based protein abundance assay, as the natural stop codons and 3ʹUTR sequences of the Ssa4, Yro2, and Hsp26 genes were replaced by identical sequences due to the C-terminal GFP tag. It is also unclear if the [*PSI*^+^]-dependent changes in the proteome contribute to the ZnCl_2_-sensitivity phenotype, or are instead a response to it. Previous studies have found that short-term exposure of yeast to ZnCl_2_ leads to oxidative stress, through consumption of low molecular mass thiols and increased production of reactive oxygen species (ROS), and also leads to the induction of genes encoding chaperones (among others) [40]. Thus, additive effects on protein abundance due to induction of stress response genes by [*PSI*^+^] [38] and by ZnCl_2_ [40] could account for some of our observations.

We assessed three environmental conditions (30°C, ZnCl_2_, and 37°C) in two genetic backgrounds (S288C and W303) with three different prion states ([*psi*^−^], [*PSI*^+^]^Sc37^, and [*PSI*^+^]^SI-str^). Although neither of the [*PSI*^+^] variants in either genetic background conferred a significant survival advantage in these environments, at least one of them (the S288C [*PSI*^+^]^SI-str^ strain) did have an apparent growth advantage over the isogenic [*psi*^−^] strain in the absence of ZnCl_2_ in that it produced larger colonies (Figure 1(a)). Intriguingly, the cells in these colonies exhibited significantly reduced survival compared to the [*psi*^−^] strain following extended incubation (Figure 2(b)). The larger colony size could potentially be due to the *ade1-14* allele present in these strains, which enables [*PSI*^+^] cells (but not [*psi*^−^] cells) to synthesize adenine; however, since we used synthetic defined medium (and not YPD) for the colony size assay, which contained an abundant supply of adenine, this is unlikely to be the cause. Additionally, the extent to which [*PSI*^+^] cells exhibited reduced survival varied considerably depending on environment, prion variant, and genetic background. Indeed, previous work has found that other [*PSI*^+^] prion variants can prolong chronological lifespan in the 74-D694 and 5V-H19 genetic backgrounds [41]. Taken together, our data demonstrate that environment, genetic background and prion variant all interact to modulate prion-dependent proteome remodeling and phenotypic outcomes. Since many environmental stress conditions have been shown to enhance rates of prion formation [10–13], and different prion variants can spontaneously arise in the population [32,42–46], the resulting phenotypic diversity could provide a mechanism for enhancing survival in fluctuating environments.

## Materials and methods

### Yeast strains and plasmids

All yeast strains used were either S288C or W303 derivatives containing the *ade1-14* allele as a colorimetric reporter for the *[PSI^+^]* prion. Construction of the S288C [*PSI*^+^]^SI-str^ SILAC strain (*his3Δ1, leu2Δ0, MET15, ura3Δ0, ade1-14-NAT^R^, Δarg4::KanMX6, lys2Δ0*) was described previously [33]. The [*PIN*^+^] status of this strain was confirmed by successful induction of [*PSI*^+^]. Strains expressing Ssa4, Hsp26, or Yro2 with C-terminal GFP fusions were constructed in [*PSI*^+^]^Sc37^[*rnq*^−^] or [*PSI*^+^]^SI-str^[*rnq*^−^] derivatives of S288C (SILAC strain, above) or W303 *(ade1-14, trp1-1, ura3::NAT^R^, leu2-3,112, his3-11,15, can1-100, MET^+^, LYS^+^*) strains obtained by protein transformation (see below). These strains were transformed with a PCR product encoding the GFP-tag and an auxotrophic marker with 40 base pairs of homology to target correct chromosomal insertion, as described previously [47]. Correct GFP tagging was confirmed by PCR with a gene-specific forward primer and a reverse primer that anneals within the GFP sequence. Isogenic [*psi*^−^] derivatives of all [*PSI*^+^] strains were generated by sequential growth (three times) on agar medium supplemented with 5 mM guanidine hydrochloride (ACROS Organics). Curing was confirmed by colony color phenotype following growth on ¼YEPD agar medium.

### Yeast growth assays

Dilutions of overnight cultures were plated onto synthetic defined (SD) agar medium with or without 5 mM ZnCl_2_. Plates were incubated at 30°C for 3 days (without ZnCl_2_) or 4 days (with ZnCl_2_) and then photographed. Colony area (pixels^2) for 161–326 isolated, single colonies was measured as previously described [48] using the ‘area’ parameter within the ‘measure’ function of the colony counter plugin for ImageJ software (Rasband, W.S., ImageJ, U. S. National Institutes of Health, Bethesda, Maryland, USA, [http://imagej.nih.gov/ij/, 1997–2014]; and Vieira, B., Colony Counter, University of Lisbon, Portugal, [http://rsb.info.nih.gov/ij/plugins/colony-counter.html]). Median colony sizes (n = 161–326) were plotted along with 5^th^, 25^th^, 75^th^, and 95^th^ percentiles and 95% confidence intervals using BoxPlotR (http://shiny.chemgrid.org/boxplotr/) [49].

### Yeast viability assays

Viability assays were performed as previously described [50,51]. Briefly, colonies on SD agar medium (with or without ZnCl_2_) were harvested in phosphate-buffered saline and incubated at 30°C for 30 minutes with 2 µM propidium iodide (Molecular Probes). Viability was assessed by flow cytometry with a BD FACS Canto II flow cytometer using the PE ‘area’ parameter. For each yeast strain, three or four independent replicates with 50,000 cells per replicate were analyzed.

### Proteomic profiling of [PSI^+^] and [psi^−^] strains exposed to ZnCl_2_

Proteomic differences between [*PSI*^+^] and [*psi*^−^] strains exposed to 5 mM ZnCl_2_ were assessed by SILAC (Stable Isotope Labeling by Amino acids in Cell culture) followed by quantitative mass spectrometry. Cells were diluted for single colonies on plates with SD-Lys-Arg medium supplemented with 5 mM ZnCl_2_ and either heavy- (Lys_4_, Arg_6_) or light-labeled arginine (20 mg/L) and lysine (30 mg/L), and then incubated for 4 days to allow for colony formation. Approximately 100 colonies were resuspended in sterile H_2_O, cell density in each suspension was determined by counting using a hemocytometer, and then suspensions of heavy and light-labeled cells were diluted to identical densities. Cells were lysed in SDS sample buffer in a Precellys bead beater and the lysates were cleared by centrifugation (16,200*g* for 20min at 4°C). About 60 µg of protein was run on a SDS-PAGE for in-gel trypsin digestion [52]. Quantitative mass spectrometry were then performed as previously described on an Impact II (Bruker Daltonics) on-line coupled to an EASY Nano-LC 1000 nanoflow HPLC (Thermo Scientific) [33]. Three independent replicates were performed and proteins were quantified using the Perseus platform (1.6.2.1) integrated to MaxQuant (1.6.2.1). The search was performed against the *Saccharomyces* Genome Database (Date stamp: 20110203). The search was configured with the following MaxQuant parameters: peptide mass accuracy 10 ppm with trypsin as the protease (K/R cleavage specificity), allowing a maximum of two missed cleavages, carbamidomethyl as fixed modification, and methionine oxidation, N-terminal acetylation, and asparagine and glutamine deamination as variable modifications. The false discovery rate was set below 1% at both the peptide and protein level.

### Protein transformation with [PSI^+^]^SI-str^ and [PSI^+^]^Sc37^ prion variants

Protein transformation to introduce the [*PSI*^+^]^SI-str^ and [*PSI*^+^]^Sc37^ prion variants into the S288C and W303 genetic backgrounds was performed as described previously [34], with some modifications. Partially purified prion particles were prepared by harvesting mid-log phase cultures, washing cells in sterile water, then resuspending in lysis buffer (40mM Tris-HCl pH 7.4, 150mM KCl, 15mM MgCl_2_, protease inhibitor cocktail mini tablet (Pierce)). Cells were lysed by vortexing with glass beads, lysates were centrifuged at 10,000g for 5 min at 4°C and the supernatant subjected to ultra-centrifugation in a Beckman Coulter Airfuge at 30 psi for 25 min. Pellets were resuspended in 1 M lithium acetate, incubated on ice for 30 min with gentle agitation, and then again subjected to ultra-centrifugation in a Beckman Coulter Airfuge at 30 psi for 25 min. Pellets were resuspended in 5 mM potassium phosphate buffer (pH 7.4) containing 150 mM NaCl and sonicated on ice for 20 seconds (20% amplitude; 10 pulses of 1 second on, 1 second off).

Cells to be transformed were first grown sequentially (three times) on agar medium supplemented with 5 mM guanidine hydrochloride (ACROS Organics) to eliminate [*PSI*^+^] and [*RNQ*^+^] prions (elimination of [*RNQ*^+^] by this treatment in these strains has been previously confirmed by lack of visible GFP foci in cells over-expressing Rnq1-GFP [48]; and our unpublished data). Cells were then treated with 100U of lyticase (Sigma) in 1 M sorbitol + 10 mM Tris pH 7.5 at 30°C for 1 hour to generate spheroplasts. Spheroplasts were collected by gentle centrifugation (400g, 4 min) and washed with 10 ml of 1 M sorbitol, harvested again and washed with 10 ml of STC-buffer (1 M sorbitol, 10 mM CaCl_2_, 10 mM Tris, pH 7.5), then collected once more and resuspended in 1 ml of STC-buffer. Spheroplasts (100 µl) were mixed with partially purified prions (final concentration ~20–40 µg), URA3 marked plasmid (~3 µg) and salmon sperm DNA (15 µg), and incubated for 30 min at room temperature. Following addition of 9 volumes of PEG-buffer (20% [w/v] PEG 3350, 10 mM CaCl_2_, 10 mM Tris, pH 7.5) and incubation at room temperature for 30 min, cells were collected by gentle centrifugation (400g, 4 min), resuspended with 150 µl of SOS-buffer (1 M sorbitol, 7 mM CaCl_2_, 0.25% yeast extract, 0.5% bacto peptone), and incubated at 30°C for 30 min. Cells were added to ~7.5 ml molten SD-URA + 2.5% agar + 1 M sorbitol (held at ~46°C), immediately mixed and plated over SD-URA agar. Colonies arising after several days were screened for prion status by color phenotype on ¼YEPD agar medium and confirmed by their ability to be cured to [*psi*^−^] following growth on medium supplemented with 5 mM guanidine hydrochloride (ACROS Organics). Subsequent passage of cells on 5-Fluoroorotic acid (5-FOA; USBiologicals) agar medium selected for cells that had lost the URA-marked plasmid used during the protein transformation protocol.

### Determination of relative protein abundance by flow cytometry

Relative protein abundance was determined by flow cytometry of strains expressing proteins with C-terminal GFP fusions as described previously [48], with the exception that cells were harvested from SD agar plates (with or without 5 mM ZnCl_2_) grown for 3 days at 30°C. For each strain, GFP fluorescence intensity was measured for 50,000 cells using a BD FACS Canto II flow cytometer and analyzed using the FITC ‘area’ parameter. For each genetic background (S288C or W303), mean GFP fluorescence was normalized to the isogenic [*psi*^−^] strain grown in the same condition.
